# The construction and characterization of the bi-directional promoter between pp38 gene and 1.8-kb mRNA transcripts of Marek's disease viruses

**DOI:** 10.1186/1743-422X-6-212

**Published:** 2009-11-30

**Authors:** Ruiai Chen, Jiabo Ding, Bin Wang

**Affiliations:** 1 College of Biological Sciences, China Agricultural University, Beijing 100193, China; 2 Guangdong Dahuanong Animal Health Products LTD, Xinxing, Guangdong 527400, China; 3 China Institute of Veterinary Drug Control, Beijing 100081, China

## Abstract

**Background:**

Marek's disease virus (MDV) has a bi-directional promoter between pp38 gene and 1.8-kb mRNA transcripts. By sequencing for the promoters from 8 different strains (CVI988, 814, GA, JM, Md5, G2, RB1B and 648A), it is found, comparing with the other 7 MDV strains, CVI988 has a 5-bp (from -628 to -632) deletion in this region, which caused a Sp1 site destroyed. In order to analysis the activity of the promoter, the complete bi-directional promoters from GA and CVI988 were, respectively, cloned into pCAT-Basic vector in both directions for the recombinants pP_GA_(pp38)-CAT, pP_GA_(1.8 kb)-CAT, pP_CVI_(pp38)-CAT and pP_CVI_(1.8 kb)-CAT. The complete promoter of GA was divided into two single-direction promoters from the replication of MDV genomic DNA, and cloned into pCAT-Basic for pdP_GA_(pp38)-CAT and pdP_GA_(1.8 kb)-CAT as well. The above 6 recombinants were then transfected into chicken embryo fibroblasts (CEFs) infected with MDV, and the activity of chloramphenicol acetyltransferase (CAT) was measured from the lysed CEFs 48 h post transfection.

**Results:**

The results showed the activity of the divided promoters was decreased on both directions. In 1.8-kb mRNA direction, it is nearly down to 2.4% (19/781) of the whole promoter, while it keeps 65% (34/52) activity in pp38 direction. The deletion of Sp1 site in CVI988 causes the 20% activity decreased, and has little influence in pp38 direction.

**Conclusion:**

The present study confirmed their result, and the promoter for the 1.8-kb mRNA transcripts is a much stronger promoter than that in the orientation for pp38.

## Background

Marek's disease virus (MDV) is an oncogenic herpesvirus, which causes a highly contagious neoplastic disease in chickens[1], and could be divided into 3 serogroups. Among them, serotype 1 could cause lymphoproliferative disease in chickens characterized by the formation of T-cell lymphomas in various visceral organs and tissues.

Based on molecular virology studies, 4 genes of MDV1 have been shown to relate to the tumorogenecity of MDV: the 1.8-kb mRNA transcript with 132-bp repeats[2,3], the 38 KD phosphorylated protein gene (*pp38*)[4], the *meq *gene [5], and *ICP4*[6]. The pp38 is a serotype 1 MDV specific protein, and there is no homolog of pp38 detected in other heresviruses of mammals and the human. The relationship between tumorigenesis and pp38 was first speculated because it was the only MDV-specific antigen detected in all non-producer MD cell lines in the mid 1980s [7,8]. Complete 1.8-kb mRNA transcripts are present in oncogenic viruses but are truncated in attenuated variants [9,10], and multiple copies of the 132-bp repeats are found in vaccine strain CVI988 or attenuated viruses compared to the virulent oncogenic strains [11,12].

Interestingly, a short fragment between *pp38 *gene and 1.8-kb mRNA family on the MDV genome contains a bi-directional transcriptional promoter sequence that controls the transcription of both genes in opposite orientations. Although the promoter sequence is only 305 bp in size, it contains the replication origin and several *cis*-acting motifs such as TATA-box, CAAT-box, Oct-1, and Sp1[2,4,13].

In the middle of this promoter region, there is a 90-bp putative replication origin of MDV genome [2,14], which shares more than 80% nucleotide identity among three serotypes of MDV, and over 70% identity with those of other α-herpesviruses [15]. When the bi-directional promoter was inserted into plasmids, however, it was found that chloramphenicol acetyltransferase (CAT) reporter gene under the control of the promoter was expressed transiently only in MDV-infected chicken embryo fibroblasts (CEF) but not in normal CEFs, speculating there was a viral or cellular factor(s) involved [16]. Our previous study showed pp38 could enhance the bi-directional promoter activity between pp38 gene and 1.8-kb mRNA, but it depends on the existence of pp24 [17,18]. Recently, CAT gene was used as a reporter to verify that the enhancement of pp38 to the promoter depends on the existence of pp24 [19], it was further confirmed by the reporter gene of Enhanced Green Fluorescence Protein (EGFP) [20].

In order to compare the activity in both directions, and investigate whether the bi-directional promoter could be divided into two active promoters, a series of CAT plasmids were constructed by using the complete or divided promoters in two directions, and then transfected to the MDV infected CEFs. These different promoters activities were analyzed in transfected cells.

There is an uninterrupted 5-bp deletion in the promoter found in CVI988, which destroys a Sp1 site. The influence of the deletion to the bi-promoter was also studied in this work.

## Results

### The complete bi-directional promoter activity in 1.8-kb mRNA direction is 15 times as that in pp38 direction

**Figure 1:**
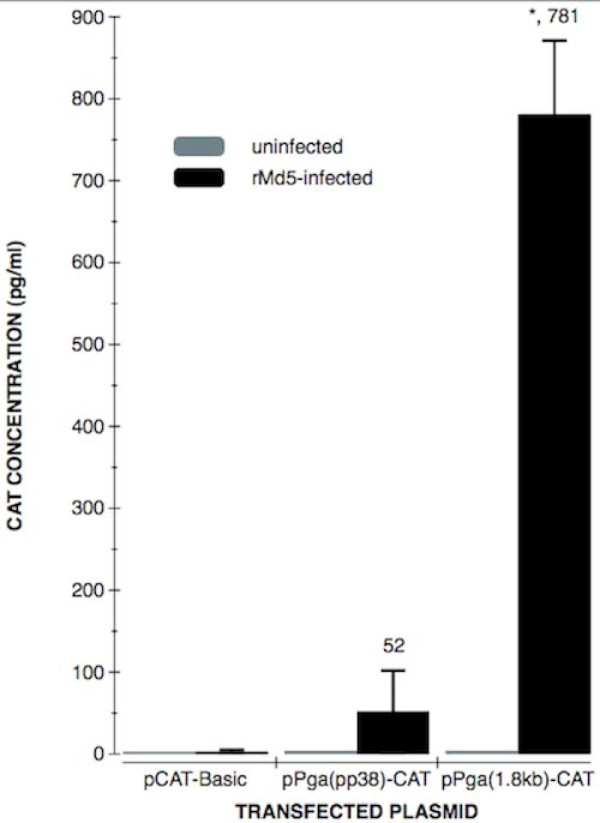
**Comparisons of CAT expression levels in uninfected CEF or rMd5-infected CEF cells transfected with plasmids pP_GA_(pp38)-CAT and pP_GA_(1.8 kb)-CAT**. Transfected cells were harvested and lysed by 3 repeats of freeze and thraw The lysed samples were analyzed for CAT activity in 96-well plate of Roche's CAT ELISA kit. Each value represents the average of at least four independent transfections and significant differences were analyzed by student's test. *, p < 0.05, compared with the pP_GA_(pp38)-CAT.

To analyze the regulation activity of the bi-directional promoter for CAT reporter gene expression, plasmids pP_GA_(pp38)-CAT and pP_GA_(1.8 kb)-CAT with the promoter in opposite directions were used to transfect CEF monolayers infected with rMd5, or uninfected CEF. The results indicated that CAT activity was at the base line level in uninfected CEF, but at higher levels in rMd5-CEF transfected with CAT reporter plasmids. The CAT activity was 15-fold higher in 1.8-kb mRNA direction than that in pp38 direction (781 ± 55.1 vs 52 ± 6.28, *p *< 0.01) (Table 1). This result indicates that in MDV infected cells, the activity of the bi-directional promoter in 1.8-kb mRNA direction is significantly higher than that in pp38 direction (Figure 1). From the data of Table 1, it indicates that MDV's infection is essential for the activity of the promoter, which confirmed Shigekane's results [16].

**Table 1:** The CAT expression levels under the complete or divided promoters in opposite directions in uninfected, or rMd5-infected CEFs transfected with a set of CAT reporter plasmids

	Complete or divided promoters in CAT reporter plasmids for transfection						
	
Transfected CEFs	Mock control pCAT-Basic	**pP**_**GA**_**(pp38)** -CAT	**pdP**_**GA**_**(pp38)** -CAT	**pP**_**GA**_**(1.8 kb)** -CAT	**pdP**_**GA**_**(1.8 kb)** -CAT	**pP**_**CVI**_**(pp38)** -CAT	**pP**_**CVI**_**(1.8 kb)** -CAT
Uninfected	3 ± 0 (3~3, n = 4)	4 ± 0 (4~4, n = 4)	4 ± 0 (4~4, n = 3)	4 ± 0 (4~4, n = 4)	4 ± 0 (4~4, n = 3)	4 ± 0 (4~4, n = 4)	4 ± 0 (4~4, n = 4)

rMd5-infected	3 ± 0 (3~3, n = 4)	52 ± 6.28 (41~60, n = 5)	34 ± 3.1 (29~39, n = 4)	781 ± 55.1 (704~842, n = 4)	19 ± 2.1 (16~23, n = 5)	54 ± 4.01 (47~68, n = 5)	635 ± 27.4 (587~700, n = 5)

The CAT expression levels were presented in concentrations (pg/mL) of the lysates prepared as in Materials and Methods. The statistics analysis was made between each pairs. For each sample, numberical figures represent following data: mean ± S.E., ranges and repeated numbers transfection assay with a given reporter plasmid. CAT activity was compared for each pairs related each factors such as CEF infection status, the complete or divided promoter and the direction of the bi-directional promoter.

### The activity of the divided promoter decreased in both dierctions

**Figure 2:**
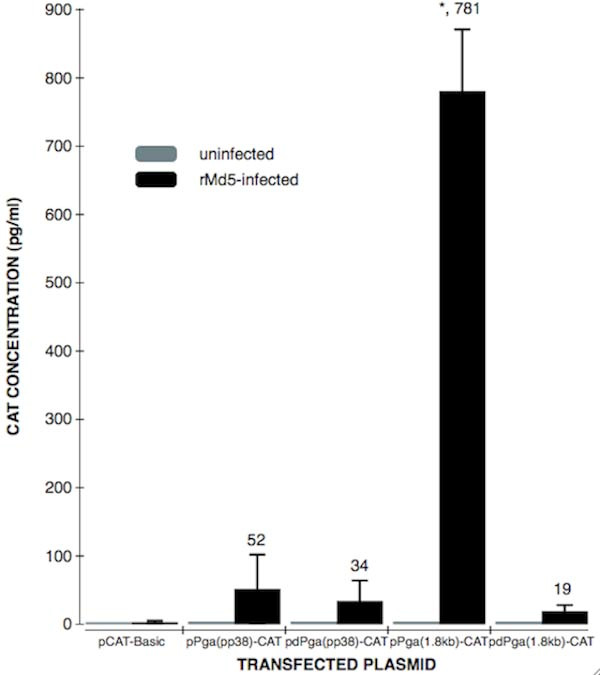
**Comparisons of CAT expression levels in uninfected CEF or rMd5-infected CEF cells transfected with the plasmids including the complete or divided promoters**. Transfected cells were harvested and lysed by 3 repeats of freeze and thraw The lysed samples were analyzed for CAT activity in 96-well plate of Roche's CAT ELISA kit. Each value represents the average of at least four independent transfections and significant differences were analyzed by student's test. * p < 0.05, compared with the pP_CVI_(pp38)-CAT and pP_GA_(pp38)-CAT.

By Compared the transfection result of pP_GA_(pp38)-CAT and pdP_GA_(pp38)-CAT, it concluded, for pp38 direction, CAT activity in complete promoter is 1.5 times higher (52 ± 6.28 vs 34 ± 3.1, *p *< 0.01) as that in the divided promoter (*p *< 0.01). Comparing the transfection groups of pP_GA_(1.8 kb)-CAT and pdP_GA_(1.8 kb)-CAT, it showed, for 1.8 kb direction, CAT activity in complete promoter is 41 times higher (781 ± 55.1 vs 19 ± 2.1, *p *< 0.01) as that in the divided promoter (*p *< 0.001). This result indicates that the divided promoters have some rudimental activity in both directions comparing to the complete bi-directional promoter (Figure. 2). It could be concluded that the intact construction of the bi-directional is essential for its entire activity.

### The deletion of the Sp1 site in CVI988 causes the 20% activity decreased in 1.8-kb mRNA direction

**Figure 3:**
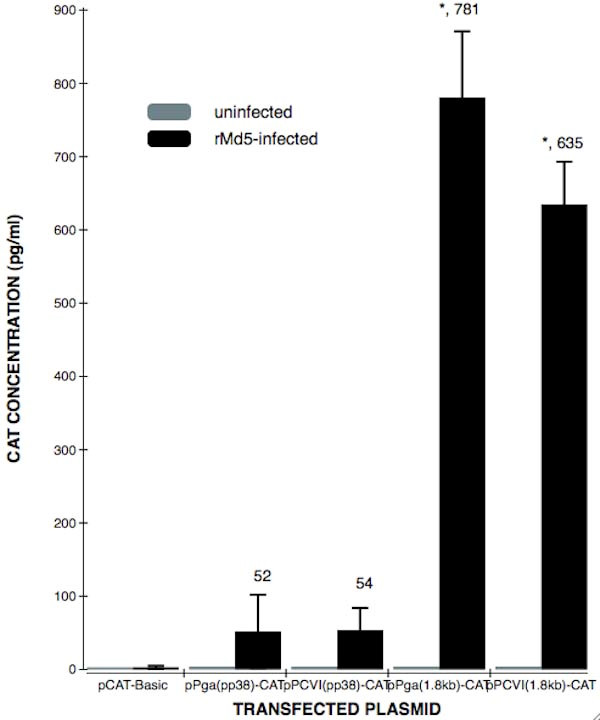
**Comparisons of CAT expression levels in uninfected CEF or rMd5-infected CEF cells transfected with the plasmids including the bi-directional promoter from GA and CVI988**. Transfected cells were harvested and lysed by 3 repeats of freeze and thraw The lysed samples were analyzed for CAT activity in 96-well plate of Roche's CAT ELISA kit. Each value represents the average of at least four independent transfections and significant differences were analyzed by student's test. *, p < 0.05.

To analyze the influence of the deletion Sp1 site on the activity to the promoter (Figure. 3), the four recombinants pP_GA_(pp38)-CAT, pP_GA_(1.8 kb)-CAT, pP_CVI_(pp38)-CAT and pP_CVI_(1.8 kb)-CAT were transfected to CEF and rMd5-CEF. For 1.8-kb direction, CAT activity by the promoter of GA origin was significantly stronger than that of CVI988 strain origin (781 ± 55.1 vs 635 ± 27.4, *p *< 0.01), but it was not significant (52 ± 6.28 vs 54 ± 4.014, *p *> 0.05) for the pp38 direction.

## Discussion

It has been recognized for many years that there was a bi-directional promoter of about 300 bp between the transcriptional start sites of the *pp38 *gene and 1.8-kb mRNA transcripts [3,4]. Beside two TATA boxes for gene transcription, the promoter contained several enhancer motifs including the Sp1, Oct1 and CAAT. In addition, a DNA replication origin and 17-bp reverse repeats were located within the promoter [5]. It had reported that the bi-directional promoter activities in two opposite orientations were regulated by common promoter-specific enhancers with a viral or cellular factor(s) induced by MDV infection. Such factor(s) could bind to a 30 bp fragment in the promoter region [16]. In our previous study, we reported that the heteropolymer pp38/pp24 could bind to the bi-directional promoter on their upstreams and regulate the promoter activity in expression of *CAT *or *EGFP *as reporter genes in transfected CEF [17-20].

**Figure 4:**
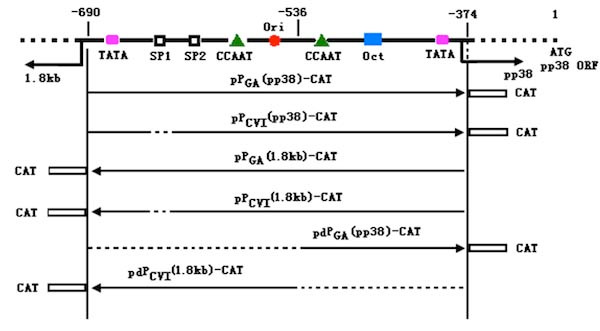
**The schematic presentation of the bi-directional promoter and the parts and directions of the promoter in different constructs**. ----, indicates the deleted region. The numbers are the sites relative to ORF of pp38 gene as described by Cui *et al *[4].

In this work, we found an uninterrupted 5-bp deletion (from -628 to -632) in the bi-directional promoter in CVI988, which destroys a Sp1 enhancer. A set of transfection showed that the Sp1 site significantly decreased the promoting activity in 1.8-kb mRNA orientation, while had little inhabitation on pp38 orientation. Analysing the structure of the bi-directional promoter (Figure 4), both of the Sp1 enhancers were in side of 1.8-kb mRNA, their enhance function may only act on the single side.

To investigate whether the bi-directional promoter may be divided into two active single-orientation promoters, we cut up the promoter from site of -536 bp concerning on its symmetrical structure (Figure 4). The divided and intact promoters were cloned into the pCAT-Basic vector, respectively. In this vector, the inserted promoter activity could be quantitative analysized according to the CAT concentration in the transfected cells. The transfection indicated the activity of the divided promoters decreased in both orientations, especially in direction for 1.8-kb mRNA. It hints the bi-directional promoter is not only an assembly by two separate divided promoters, but also organized as a whole. Its entire activity is interrelated with the intact structure.

## Conclusion

It was reported that CAT-activity expressed under the bi-directional promoter in the direction for 1.8-kb transcripts was significantly higher than that from the pp38 direction [16]. The present study confirmed their result, and the promoter for the 1.8-kb mRNA transcripts is a much stronger promoter than that in the orientation for pp38.

## Methods

### Materials and reagents

pUC18 vector, T4 ligase, and all the enzymes were purchased from TaKaRa Biotechnology Co., Ltd (Dalian, China). Lipofectamine™ was purchased from Invitrogen (Beijing, China); plasmid purification Mini Kit was from Qiagen (Shanghai, China); pCAT-Basic vector was from Promega (Beijing, China); CAT ELISA detection Kit was from Roche (Shanghai, China); SPF chicken embryos were from SFAFAS Company (Jinan, China).

### Cells and viruses

MDV rMd5 was rescued in culture from five cosmids containing a whole genome of parent virus Md5, which was kindly provided by Dr. Reddy S [21]. This rescued rMd5 has a clear genetic background and predictable growth rate in CEF cells after its transfection. Eight distinct virulent MDV strains were used as templates to amplify the promoter regions: virulent strains (GA [22] and JM [23]), very virulent strains (Md5 [24], G2 ([25], and RB1B [23]), very virulent plus strain 648A [26] and vaccine strains (CVI988 [27] and 814 [28]). All the strains were kindly provided from Dr. Cui Z. Z. These viruses were propagated in primary chicken embryo fibroblast (CEF) cells and inoculated with MDV-infected CEF at a 10:1 of CEF:virus-infected CEF ratio. The cell pellets were used for extraction of total genomic DNAs by proteinase K (Merck Co., Beijing, China) and phenol solutions as previously described[17].

### Construction of recombinant plasmids expressing CAT gene under the control of different promoters

Construction of the bi-directional promoter was made by the use of a series of primers synthesized for the complete or divided promoters by PCR. For example, the promoter P (pp38) in pp38 transcriptional direction with forward primer: 5'-AAGGTACCGAGCATCGCGAAAGAGAGA-3' (bases -690 to -671, relative to pp38 gene ORF, plus a *Kpn*I site, underlined); and reverse primer: 5'-GTGAGCTCTCGAGGCCACAAGAAATT-3' (bases -393 to -374 plus a *Sac*I site, underlined). All the primer sequences and the putative fragments are listed in Table 2. In PCR amplification, two pairs of primers F_pp38_, R_pp38 _and F_1.8 kb_, R_1.8 kb _were used with genome DNA of GA and CVI988 strains, respectively. The divided promoters were amplified with only the template of GA. All the PCR fragments were sequenced before inserted into the *Kpn*I/*Sac*I sites of pCAT-Basic vector (Promega, Beijin, China). In the recombinant plasmids, pP_GA_(pp38)-CAT, pP_GA_(1.8 kb)-CAT and pP_CVI_(pp38)-CAT, pP_CVI_(1.8 kb)-CAT, CAT was expressed under the regulation of the promoter in opposite directions. The diagram for different promoters and recombinants is shown in Figure 4.

**Table 2:** Primers used to generate a serial of plasmids to validate the activity of the promoter

Primer	Sequence(5^1^-3^1^)	**The sites opposite to the ORF of pp38**[4]	Restriction enzyme sites	Fragment generated/bp
F_pp38_	AA**ggtacc**GAGCATCGCGAAAGAGAGA	-690~-671	*Kpn*I	322
R_pp38_	GT**gagctc**TCGAGGCCACAAGAAATT	-393~-374	*Sac*I	
F_1.8 kb_	AA**gagctc**GAGCATCGCGAAAGAGAGA	-690~-671	*Sac*I	322
R_1.8 kb_	GA**ggtacc**TCGAGGCCACAAGAAATT	-393~-374	*Kpn*I	
F(d)_pp38_	TTT**ggtacc**GTTCGCACCAGAGTCCA	-536~-519	*Kpn*I	168
R(d)_pp38_	GAA**gagctc**GAGGCCACAAGAAATT	-393~-374	*Sac*I	
F(d)_1.8 kb_	AA**gagctc**GAGCATCGCGAAAGAGAGA	-690~-671	*Sac*I	161
R(>d)_1.8 kb_	AAA**ggtacc**GCCGAGGTGAGCCAATC	-552~-535	*Kpn*I	

### Transfection of the CAT expressing recombinants to uninfected CEF and rMd5-CEF

Primary CEF cultures were prepared in a 60-cm2 flask until cells formed a monolayer and infected with rMd5-CEF stocks of 1×10^5 ^plaque form unit (PFU). The infected cell cultures were incubated for 3-4 days until cytopathogenic effect (CPE) was appeared in the monolayers. The MDV-CEF monolayers were trypsinized and the viable cell number was determined. One part of the MDV-CEF suspension was mixed with two parts (by cell number) of fresh secondary CEF suspension and placed into 35 mm dishes (1×10^6 ^cells per dish). To prepare the secondary CEF monolayers, 1×10^6 ^cells were seeded into 35 mm dishes until cell monolayers formed 18-24 h later.

Transfection was carried out 18 h later when the secondary CEF monolayers were formed. Transfection of each recombinant plasmid DNA was performed by using LipofectAMINE™ reagent according to the manufacturer's instructions. Briefly, 2 μg plasmid DNA and 4 μl LipofectAMINE™ reagent were added into two separated polypropylene tubes with 100 μl of DMEM medium free of serum and antibiotic. These two solutions were mixed and incubated for 45 min at room temperature and then added into another 800 μl DMEM. A total of 1 ml of the transfection solution was carefully poured onto the cell monolayers in a 35 mm dish. After 8 h, 1 ml of complete medium with 10% bovine fetus serum were added to the transfected cell monolayers. All dishes were maintained at 37°C in a CO_2 _incubator. The expression of CAT was determined 48 h after transfection. The transfection on uninfected CEF was carried out as well as control.

### Determination of CAT activity in transfected CEFs

Two days after transfection with plasmids pCAT-Basic (control), pP_GA_(pp38)-CAT, pP_GA_(1.8 kb)-CAT, pP_CVI_(pp38)-CAT, pP_CVI_(1.8 kb)-CAT, pdP_GA_(pp38)-CAT and pdP_GA_(1.8 kb)-CAT, the transfected CEF were harvested and resuspended in 500 μl lysis buffer (0.25 M Tris-HCl, pH7.0) per 35 mm dish. After 3 freeze-thaw cycles, samples were centrifuged for 5 min at 10,000 rpm. Aliquots (200 μl) of the supernatants were added into wells of 96-well ELISA plates to test CAT activity using CAT ELISA Kit (Roche, Cat.No.1363727). The concentration of the CAT in the lysates was measured using a calibration curve of known specific standards according to the manufacturer's instructions. Five replicates of transfections were carried out with 6 different CAT plasmid DNAs in each of rMd5-CEF or uninfected CEF cells. The significant differences among the groups were analyzed by student's test. The CAT activity in the pCAT-Basic transfected samples were also determined and analyzed as described.

## Competing interests

The authors declare that they have no competing interests.

## Authors' contributions

RC and DJB designed and performed experiments; DJB and BW analyzed the data and wrote the manuscript.
